# Philosophy of Spirits
*Philosophy of Spirits in relation to Matter. By A. N. Burnett, M.D. 1850.


**Published:** 1851-04-01

**Authors:** 


### Art. V.—PHILOSOPHY OF SPIRITS.*

Dr. Burnett is a bold man. He has risen to an altitude unexampled in the records of metaphysical science. In vain do we attempt to follow him through the realms of space. Occasionally we are fortunate enough to obtain a glimpse of the philosopher during one of his exalted flights; but, alas! we soon lose sight of him, and are compelled to leave him in his own elevated sphere.

It required more than an ordinary amount of moral courage to admit oneself related by the closest ties of consanguinity to a work in which an attempt is made to subvert nearly all the recognised truths of philosophy.

We are startled by the daring character of the assault. But we presume the author, in writing his work, kept before him the following observations of the illustrious founder of the inductive philosophy, who

* Philosophy of Spirits in relation to Matter. By A. N. Burnett, M.D. 1850.

says, "The sciences have been much hurt by *pusillanimity*, and the *slenderness of the tasks* men have proposed themselves."

Dr. Burnett has been careful not to expose himself to such an imputation. We subjoin an outline of what the author proposes to establish in the work before us. It is his object to prove that heat and electricity are distinct entities, co-ordinate in rank and of an immaterial or spiritual nature—that other *properties* manifested in the organic and inorganic kingdoms are also spiritual and immaterial, although varying as to their relative degree of power—that form, consistence, colour, taste, &c., as qualities, and electricity, light, motion, life, &c., as phenomena, are the result of the union of these kinds of entity, with others which are material—that these entities, viz., heat and electricity, were in a conjugate state as constituting created matter—that imponderable matter and force are mere effects which follow the union or application of the two entities, heat and electricity, in different ways—that motion is a phenomenon resulting from the action of the spirits of heat and electricity upon matter—that the prevalent idea of spirit being circumscribed within that of a *conscious being* is incorrect—that mind is a mode of action by which the characters and qualities of everything around are depicted—that the spirit of electricity closely resembles that which goes to form mind in that particular *power* they both possess of spanning objects *immeasurably* distant—that mind cannot be separated from the *spirit* of life, and both result from the same spirit—that mind and instinct are identical in their nature—that the phenomena of the galvanic battery, the transformation of water into steam or ice, the globularity of bodies, and the production of inorganic bodies, are solely dependent on the spirits of heat and electricity for their existence, and the phenomena which they manifest—that all created worlds are alike composed of matter and spirit—that the gravitation of matter, or the ponderosity of material bodies, is solely referrible to the influence exercised by the spirit of electricity—that the faculties and feelings have their root in the sentient power of the nervous system; and the attributes of mind, their root in the motive power of that system—that the loss of the power of the *will* over any particular desire constitutes, in a true psychological sense, an unsoundness of mind—that it has been shown by morbid anatomy, that the various losses on particular points have been accompanied by more or less extensive lesions of the brain—and that no plea of insanity in criminal cases should exempt a man from punishment on the ground of his not being able to distinguish right from wrong, so that he is able to understand the true characters and uses of things after they have been explained to him intellectually.

It will be our endeavour to establish that Dr. Burnett has explained his hypotheses, (according to our comprehension), *ignotum per ignotius.* He has attempted to elucidate that which is unknown by a reference to a something more unknown.

It would appear that the theory of spiritual entities has originated in a difficulty on the part of the author to conceive material entities to exist without the property of *ponderance;* and, from a mistaken supposition that the qualities or properties, accidents, and modifications of matter, are something distinct from matter itself. These errors, which constitute the fundamental principles on which Dr. Burnett's work is based, covertly intermingle themselves throughout every part in which he attempts to elucidate natural and experimental philosophy.

The first point which claims our attention in reference to the volume under consideration is the important question raised by Dr. Burnett, whether *ponderosity* is the proper criterion by which *material* can be contradistinguished from *immaterial* entities?

Hear the author:—

"To say that light, heat, and electricity are imponderable bodies, is not only negative and indefinite, but, strictly speaking, it is not sense, if material bodies are intended to be expressed by this term; and we know of no other to which the term can be applied. There is no material body that is actually imponderable; and, when any such matter can be shown to be deficient in this quality, it is no longer material to common sense, neither is it so in a true philosophical sense; for the weight of a body is the amount of attraction the immaterial spirit of electricity exercises on that particular body as a created body; and this must be more or less existing, or the body could not be retained on the surface or in the atmosphere of our earth. For this cause it is that spirit cannot be retained or confined to the earth, or to any particular part of space; and, therefore, all material substance, whether uncombined or united with spirit, as in the visible creation, has this quality of ponderosity given to it."—p. 51.

It is clear that Dr. Burnett fails to recognise what are, and what are not, the *essential* properties of matter. That peculiar law which pervades the solar system, termed *gravitation,* and to which the ponderosity of material bodies is attributable, is not an *inherently essential property of matter.* The ponderosity of bodies cannot, therefore, be the proper criterion by which to distinguish an immaterial from a material entity. He affirms that "there is no material body which is actually imponderable." To which class, then, would he refer *odours,* given off and diffused through the atmosphere by odoriferous substances? A grain of musk has been known to diffuse the characteristic odour of that substance through the air of a large room for twenty years without any appreciable diminution of its weight. Is odour, then, a material or immaterial quality? It is self-evident that the odour could not affect the olfactory nerves without the diffusion of some portion of its substance through the air; while the diffusion must characterize the presence of material particles having no weight, as the substance from which they proceed loses none in any way detectable.

Thus, there are in nature parallel proofs, that ponderosity is not the *essential* criterion by which a *material* can be distinguished from an *immaterial* entity.

We allude, *en passant*, to the extreme divisibility of many substances capable of being recognised by *their colours* only, and without which the most delicate test could not detect them.

We have, however, to substantiate the extraordinary statement, we think, for the first time promulgated, that *ponderosity* is not absolutely essential to the existence of matter. This *property*, on which Dr. Burnett bases his theory of immaterial entities, affirming "that there is no material body actually imponderable," is entirely dependent on the law of gravitation, the operation of which decreases in the inverse ratio of the square of the distance. Matter may exist apart from the property of *weight—i. e.*, gravitation, and yet be matter still. If *gravitation* had been an absolutely essential property of matter, the same body would always retain its original quantity of gravitation in every position in which it might be placed, and no such property as repulsion could have existed.

The same remark will apply to the opinion, "that the weight of a body is the amount of attraction the immaterial spirit of electricity exercises on that particular body, as a created body," &c.; this motion destroys the "spirit of heat," which causes repulsion, so much so, that the body possessing this amount of electricity, will always retain its original quantity in every position in which it might be placed.

But this hypothesis, taken in its fullest import, destroys itself; for electricity is supposed to be *spiritual*, on the ground that *gravitation* is not associated in the idea of its existence; and yet we are now gravely informed, that the weight of a body—*i. e.*, its gravitation, is the actual amount of *that immaterial entity*, which before was predicated as an entity without weight! By what *modus existendi* is this spiritual entity without the properties of matter (weight, &c.) at one time, and at another possessing and communicating such properties? It must be admitted, that whatever is adduced as an exception to every other created entity, ought to be substantiated by such proofs as will admit no doubt of its existence.

The same reasoning, by which gravitation is proved not to be a property absolutely essential to matter, will also show that the "amount of attraction exercised by the immaterial spirit of electricity," cannot constitute the criterion by which we denominate one entity ponderable, and another entity imponderable.

If gravitation, or the weight of created bodies, depends on the spirit of electricity, and if this spirit be a real entity, then ponderosity, or gravitation, as its cause, must be an entity of the same nature—viz., spiritual, immaterial, and independent.

But "form, consistence, colour, taste, &c., as qualities; and light, electricity, motion, life, &c., as phenomena, are stated by the author, not only to owe their existence, but to be dependent on the conjunction of materiality, heat and electricity.*

It is evident, from this statement, that electricity is supposed to pervade *universal nature*, that gravitation, and ponderosity, its effect, are properties absolutely essential to all created matter. And this must be so, if all material entities have originated by the union of heat and electricity, and if ponderosity be dependent on the latter—an effect always being co-equal and co-extensive with its cause.

The supposition that electricity produces the phenomena of gravitation in *all created matter*, will at once be seen inconclusive, by a demonstration of its limitation to the solar system. So will the hypothesis which assigns a positive immaterial existence, a creative, and sustaining energy, to heat and electricity. The following quotation defines clearly the author's opinion on gravitation, and the active agency to which it is referrible:—

"By electric immateriality it is that the earth receives the power we term *centre of gravity*, and that of revolving on its own axis, so that, as if it really turned a material axle, it holds the globe in obedience to certain and unalterable movements. These movements are as much the sensible effect and evidence of its real existence in the boreal and austeal poles of the magnetic axis, as colour in material bodies is a sensible evidence that the spirits of heat and electricity have been brought to bear upon them."—p. 81.

Thus, the motion of the earth, in addition to the property of gravitation, is ascribed to electric immateriality. The author, however, amalgamates the agency of magnetism in affording a more satisfactory explanation of his opinion on the earth's motion.

"The effect," he observes, "of the spirit of electricity upon all bodies containing iron, is to produce what is called the magnetic power in those bodies when placed in a particular position. This power is not conveyed to them in the same manner as the spirit of heat would be; but when those bodies are placed at right angles to the line of direction of the electric spirit. And this fact, first discovered by Oersted, puts

* Vide Preface, and page 11.

us in possession of the fundamental rectangular force—a force unlike any hitherto discovered, by *which the earth is made to revolve on its own axle,* while an extension of the same power would give the heavenly bodies a rotatory movement in their own orbits. Thus, a stream of the electric spirit passing through the centre of a circle whose plane is perpendicular to the current, the direction of the electric spirit will always be in the tangent of the circle, or at right angles to its radius. And while these spirits help to draw the heavenly bodies in their orbits round the centre of attraction, they also serve to maintain all material substances intact upon the earth."—p. 81.

On the supposition that the electric spirit produces the phenomena of gravitation through *universal nature,* a law is predicated with respect to electricity, from which divarication is impossible. Any exception, therefore, to a fact of which universality, or absolute essentiality, is predicated, destroys it. It shows the fact to be *universal* and *not universal* at the same time, which is a contradiction. The very term, universal, also excludes the idea of intention, or remission, and consequently, variation or exception.

To the notion that electricity and electro-magnetism produce the phenomena of *universal gravitation,* motion, ponderosity, &c., there are many exceptions; and many facts to which the author himself adverts, that show its inconclusiveness. We shall point these out as we proceed.

According to the constitution of nature, the detached particles of matter gravitate towards the centre of the earth; and the earth, with its appendages, gravitates towards the sun. By the same mode of reasoning, we have satisfactory evidence, that all the orbs which compose the solar system, feel a proportionate impression; even if we take into the account the eccentricities of the comets, which seem to be the most erratic of all the heavenly bodies with which we are acquainted.

But though the various bodies of the solar system thus gravitate towards their common centre, it will not follow that the whole system when taken in an aggregate point of view, gravitates towards any other system in the universe. It will indeed admit of much more probable evidence, that no such gravitation either does or can exist. For, if gravitation can exist in the solar system towards any other system whatever, it will be impossible to assign any satisfactory reason why the branches of different systems continue apart from one another; and to say what has prevented that contact, which necessarily results from the direct action of gravitating bodies.

Dr. Burnett is however of a contrary opinion, and adduces evidence to show that all created worlds have been made, are sustained, and will be destroyed, through the agency of the same material and immaterial entities.

On this point he remarks:—

"I think it may be very strongly inferred that the two entities (heat and electricity, which enter into the construction of the materials of our globe, and which are made to perform the various phenomena that are attached to it, have been made to enter into the construction and to cause the phenomena, of other worlds besides our own. And this may be deduced from the uniformity that pervades and characterizes the whole of the phenomena of the solar system. In this case it will be necessary to bear in mind the argument that has been used to show that the sun in the centre of our system is a composite body, made up of the two kinds of entities I have already stated there is so much reason to believe exist."—p. 86.

The same evidence by which Dr. Burnett attempts to show the *universal extent* of the two entities, heat and electricity, establishes the supposition, that gravitation and ponderosity equally pervade created matter.

We are, however, of the opinion, that gravitation depends upon the local circumstances of time and place, and that if it so pleased God, it might be separated from, or be non-existent, without the annihilation of matter, or its essential properties, necessarily ensuing.

The consequences which would follow the supposition, that either gravitation or electricity is a property of *universal matter*, have been entirely overlooked by the author—a property that must necessarily bring together, instead of preserving distinct, the various systems.

That the worlds, which Deity has fixed in the immensity of space, are infinite in their extent, Dr. Burnett will not, perhaps, affirm; the exterior systems can, therefore, have nothing to prevent them from falling immediately upon those which are most contiguous. The second, after having overcome the first, must act in the same manner towards its neighbouring system, till that also sinks in ruin; and thus destruction must press upon destruction, till those worlds which now adhere to their respective systems, are reduced to a state of confusion, and blended together in one chaotic mass.

The existence of the various systems with which we are surrounded, and of which we make a part, proves that no such effects as

"The wreck of matter, and the crash of worlds"

have taken place; and hence we may reasonably presume that no such extent of gravitation, or electricity, does exist. And, since the general convulsion of the universe would inevitably ensue, if such an operative power were to pervade created matter in its entirety, the order which subsists throughout the universe indubitably proves that no such property as gravitation, or electricity and ponderosity, according to the views of Dr. Burnett, can diffuse its influence through *universal nature,*

nor probably reach beyond the different systems to which its influence is confined. Pope gives us an exponent of the same idea when he says—

> "The general order, since the world began,
> Is kept in Nature, and is kept in man."

From these principles, it is fairly to be inferred that, although gravitation is so closely interwoven with the whole system of matter in all the forms into which it has been modified, as to be naturally inseparable from its minutest parts, yet, that it is confined in its operative influence: and that, as it is local and circumscribed in its action, it cannot, in the strictest and most philosophical sense of the word, be an essential property of matter. The same reasoning applies to *electricity.*

The opinion which the author has expressed relatively to the origin of aeorolites and meteorolites, invalidates, instead of supports, the general doctrine sought to be established.

> "Coming (he observes) as there is the greater reason to suppose they do, from other planetary bodies floating in the same system, they must be regarded as, and placed amongst, the rational proofs to be brought forward of the identity that exists in the primary and uncombined elements of our own with other created worlds."—p. 88.

By this supposition, gravitation, or electricity, its substitute, pervades systems of matter other than the solar system—the absurdity and fatal consequences of which have been pointed out.

The arguments adduced to show that gravitation does not pervade *universal nature,* clearly prove that ponderosity, its effect, cannot be a criterion by which to distinguish a material from an immaterial entity. If systems of matter can exist uninfluenced by gravitation, then that property which Dr. Burnett regards as an immutable criterion of its nature, is reduced to a nonentity; leaving a substance having solidity, magnitude, and figure, and yet without weight.

There are many instances with which we are acquainted, in which gravitation may be lessened in its influence, suspended in its power, and partially destroyed; while the matter itself in which it inheres, retains all its essential properties, and undergoes no real change.

The extraordinary influence of electro-magnetism is well known; and yet, strange to say, Dr. Burnett alludes to this fact (p. 83) in support of his views.

To establish a law, rule, or principle, in connexion with material objects, we must generalize from individual facts, and if we find that they are invariably true, we may infer a general fact—*i. e.,* a general law, or general principle.

If electricity be the cause of ponderosity—*i. e.* of gravitation, how is it that it possesses the power to suspend the influence occasioned by itself, in a bar of magnetized iron? Here is an exception—a fact which disproves the uniformity of the general fact, and therefore destroys instead of supports the theory which it is designed to establish.

From the arguments which have been adduced, it is evident that gravitation can only be an affection of matter, existing in relation to time and place, and by no means an essential property of that substance in which it is presumed to inhere.

What the physical nature of gravitation is, we do not with precision know; but of this we are fully assured, that it is an universal affection of matter, through which all material bodies are disposed to approach towards each other, and their respective centres; while ponderosity, which depends upon this affection of matter, demonstrably shows that this cannot be the absolute test by which to distinguish an immaterial from a material entity.

It would be foreign to the object which we have in our analysis of Dr. Burnett's work, or we could point out the highly important and interesting relation which the bearings of this subject have in affording a solution of that complex assertion which St. Paul has made—" *There is a natural body, and there is a spiritual body.*"

Suffice it, however, to observe, that the human body, having the power of gravitation in its present state in common with all sublunary beings and things, may with much propriety be termed a *natural body;* and that by being divested of this quality, which we have shown not to be an essential property of matter, will then, doubtless, become what St. Paul denominates a *spiritual body.*

We are now prepared to enter upon an analysis of the argument adduced to show, in the language of Dr. Burnett, the real existence of two separate and created kinds of entity in the universe, by the union of which we behold every created thing—these are heat and electricity.

" The laws of nature, and the influence these laws exert upon the matter that surrounds us," being first noticed by the author, he goes on to observe that natural philosophy—

" Has on no occasion proved them to be the result of anything inherent in matter alone; and if, therefore, they are not the effect of anything that is material, it is the more probable they are the result of something that is immaterial, and that does not partake of the character of material substance; and I think there is more than common evidence to show that these forces, or laws, as they are called, are the result of some *substantive* and distinct, though immaterial and dependent spirits while acting upon material matter."—p. 9.

These he believes to have been created distinct, though necessary to be brought in union, to make them evident to the senses.

"God," he says, "created other substances that were of an immaterial nature, and by these He brought materiality into light, order, and beauty, and made them manifest to our senses."

Hence, the present system of natural philosophy is repudiated as fallacious and problematical; while another of the author's own construction is raised to the rank of postulates, and axioms in admitted truths. Thus he affirms:—

"That there are two distinct and characteristic kinds of substances, both alike as entities, but totally different and opposite in their nature; which, by a power inscrutable to us, the Creator has made to act the one upon the other in the production of all those qualities we observe to characterise the natural bodies around us, and of all those phenomena hitherto termed the laws of nature; and that these two very different substances are found to exist in the universe under two distinct forms: 1st, in union, as we find them locked up together in the construction of every natural and created body, in which state of combination they produce the qualities of form, size, colour, consistence, taste, &c.; and 2ndly, in a separate and uncombined state, as we find them existing in the atmosphere, whence they are taken as they are required to form new synthetical unions in the construction of vegetable and animal structures, as well as to produce the phenomena of light, heat, and electricity, by which these structures are partly sustained. In this uncombined state they also act upon created matter to the production of the great forces necessary for sustaining the different celestial bodies in their relative positions and motions."—pp. 10, 11.

Regarding our relation to the external world, we maintain that our knowledge of heat and electricity,—the two grand immaterial entities, to which Dr. Burnett ascribes the existence of matter, its laws, and phenomena—have been derived from these sources, and the sensations to which they give rise.

But if matter be necessary to the existence of heat and electricity—which cannot be denied—they can have no *real existence*, and therefore can only be relative terms.

If heat and electricity be immaterial entities, they must exist abstracted from matter, its modes and combinations; for whatever exists positively, must have a being before it can possibly combine; and what has a positive existence, must be independent of all combination. But if heat and electricity exist abstracted from combination, they can have no connexion with matter, for matter itself must be the result of combination; and to suppose anything to have a *necessary* connexion—to say nothing of causation—with matter, from which it is possible to exclude the idea of combination, is a contradiction.

If these immaterial entities be limited in the nature of their existence, and confined wholly to matter, for an evidence of that existence, matter must form those lines, beyond the boundaries of which they cannot pass. To admit anything to have a positive existence, which is not independent, is a contradiction in terms; and to suppose the independence of those entities, which cannot pass the boundaries of matter, while nothing physical obstructs such power, is to suppose them to be independent and not independent at the same time.

As a contradiction must ever be inadmissible, it follows, that heat and electricity, whatever they may be, can have but relative modes of existence; relative modes of existence must always be dependent on those objects to which they are indebted for their being, and can no longer exist than while they are excited by a foreign power. If matter be the only medium through which these immaterial agencies operate, they can of themselves exert no influence.

If heat and electricity possess self-operative powers, which the hypothesis of Dr. Burnett implies, they can be no longer dependent on matter; but to abstract matter from these immaterial entities, and to suppose the latter to exist after this abstraction, is to attribute powers which are precluded, by the very nature of their existence.

Whatever has but a relative, must exist in its manner different from that which has a positive existence. That which is of itself but a *mere quality*, can not, *philosophically speaking*, have modes and accidents. Nor can any thing which is but *a quality*, have any *quality* which depends upon it for its existence. How then can heat and electricity, which are but qualities of matter, or more properly speaking, modifications of matter, impart solidity, magnitude, and figure, the essential qualities of that substance? The illustrations which Dr. Burnett adduces in support of his opinions, destroy them at every step. For if heat and electricity be but qualities of matter, by the latter understanding that substance in which *solidity*, *magnitude*, and *figure* inhere, they cannot have any *qualities*, as colour, taste, temperature, &c., which depend upon them for their existence.

To suppose any one quality to depend upon another *mere quality* (except primary qualities, which are totally unknown) for its existence, is to make the former quality to commence cause, and to make the latter dependent quality to derive from the former a certainty of existence, which the former does not possess, and which therefore it cannot communicate.

We cannot discriminate a material from an immaterial entity, by the properties of temperature, colour, motion, life, &c.; for temperature is variable, colour uncertain, motion not natural to matter, and life dependent on organization; it is therefore evident that it is in such properties only as solidity, magnitude, and figure, that the idea of matter can possibly inhere.

As heat and electricity have no relative dependent qualities, they can be but qualities in themselves, qualities of a substance, the essential properties of which clearly demonstrate their materiality.

If the nature of substances were not denominated from their own essential properties, it would follow that these *essential* properties were *not essential,* which is a contradiction. But if the substance be denominated from its essential properties, and these essential properties are known, we then have, from our knowledge of the essential properties, all that knowledge of their substance which is within the reach of possibility, supported by the unequivocal evidence of demonstration.

If heat and electricity be immaterial, we would ask—how can they, by inhering in any common substance (which cannot be denied), acquire from that substance a nature, whose qualities are totally distinct from their own? If they can, then these qualities are not necessary to the existence of that substance, because their nature is distinct; if not, these qualities, viz., heat and electricity, must be material. If these qualities be not essential to the existence of that substance in which they are supposed to inhere, they may be separated; and if separated, we would again ask—what idea can we form of their *abstract existence?* And what idea can we form of that substance from which they are abstracted? To suppose this *substance* to be *matter,* is to make *heat* and electricity not to be necessary to the existence of *solidity, magnitude,* and *figure,* the essential properties of matter; and to suppose *them* to be *immaterial,* is to suppose them immaterial, while every property is abstracted from which spirituality is denominated: that they are spiritual entities without spiritual powers—and that they are immaterial and not immaterial at the same time.

Contrary, however, to this reasoning, the author maintains that materiality could not be brought into actual existence, without an union with immaterial agents altogether distinct from those material elements.

"The very method," observes Dr. Burnett, "by which material bodies are made to act upon our senses, in the first instance to convince us of their real existence, could never be accomplished by material substances of a like nature to themselves, and unassisted by other substances of a different nature, which are, in fact, created spirits; and, accordingly, as we find the same wonderful contrivance resorted to by the Creator in bringing into sensible existence the whole living creation, so here, in the first instance, we behold Him, by means of a power which *He* alone possesses, bringing immaterial substances to bear upon those that are of a material nature, by which means they are brought out of the simplicity of uncombined chaos into beauty, order, and consistence."—p. 12.

In opposition, therefore, to reasoning, so evidently illogical and absurd, we affirm, that, when the nature of two substances is incompatible, by a positive disagreement in their essential qualities, they cannot be united without some proper medium.

If heat and electricity be immaterial entities, they must have all the properties which are essential to spirituality; to suppose otherwise, is a contradiction; and whatsoever has the properties of *immateriality cannot occupy space.*

But Dr. Burnett affirms differently:—

"It is from the great difference," he observes, "in the visible appearance of the heavenly bodies, that I am led to suppose the immaterial substances have not only different qualities, and also relative degrees of power, but that they possess also a power of occupying all space."—p. 89.

To suppose immaterial substances to have the power of occupying space, without including the idea of extension, is a contradiction. And that substance, of which extension can be predicated, must be material. And whatever occupies or fills empty space, must have dimensions. But to attribute dimensions to the *immaterial entities*, heat and electricity, whose existence can only be ascertained by those qualities which must necessarily be *immaterial*, and which qualities must be essentially necessary to the existence of these entities, is to suppose these entities to be *immaterial*, while we have no conception of such natures, and while the only qualities which denominate and establish their existence, exclude the idea of *immateriality* from our conceptions.

And to suppose, under these circumstances, the entities, heat and electricity, to be immaterial, is to admit the idea of *immateriality*, upon the evidence of *material qualities*, by which the supposition is destroyed. We are, therefore, bound to conclude, that heat and electricity are not the positive immaterial entities which Dr. Burnett has thought fit to regard them.

The fallacious theory of Dr. Burnett has originated in part from a total disregard of the primary cause on which the variable or secondary qualities of matter depend.

Numerous and extraordinary as these are, we have no hesitation in referring them to the modification of matter, and not to the presumed *modus operandi* of immaterial agents.

Brittleness, elasticity, electricity, magnetism, chemical affinity, colour, &c., are but the results of certain modifications of matter.

Matter, under every form, can be but matter still; and whether we choose to denominate some portion, or some property of it, as being immaterial or not, its real essence can be by no means altered by this distinction. If it be matter, it must, in all its states, have all its properties; and by all the modifications which it is capable of undergoing, it can acquire nothing new.

Even the author cannot reconcile or explain every material phenomenon by the unbounded power of his immaterial spirits. On alluding to this, he observes:—" Like the material matters of the universe, the action of one, two, or more, upon each other, is productive of the most unaccountable difference in the outward appearance of bodies, which the laws of synthesis have not, in our present state of knowledge, attempted to explain."—p. 129.

Synthesis can never explain what is dependent on modification. Electricity, temperature, or heat, being dependent on the modification of matter, another source of evidence arises to expose the fallacy of the *immaterial theory.*

To suppose that the *mere modification* of any *entity* will enable that entity purely, from this modification, to be capable of producing effects —such, for example, as the transformation of the qualities of heat and electricity into spiritual entities, with which all the parts of the body modified have no relation, is to suppose that it receives an additional power, which nothing but modification can communicate; while modification itself can have no existence but what it derives from the parts so modified, and which of themselves can possess no such power, which is a palpable contradiction.

All bodies, under every modification, must be formed of parts, and though in coalescence, they are still the same; and if a power to produce the immaterial entities, heat and electricity, does exist in matter, it must result from the particular arrangement of its component parts. Every *whole* must be formed of those parts which are necessary to its existence; and, to conceive that the immaterial entities, heat and electricity, can result from any modification of these parts, is to conceive that the whole possesses a power, that *all* and *every* part of which it is composed are totally destitute; in fact, that the whole, which is formed only of certain parts, is capable of communicating what it neither possesses nor has received; or, in other words, that it is capable of producing *immateriality*, and yet incapable at the same time.

An assemblage of atoms may produce an increase of magnitude. A modification of parts may produce a change of figure. A new disposition of surfaces may produce different sensations, and variously affect the organs of vision; but all the changes which matter is capable of undergoing are only capable of *enlarging* or *lessening* the extent of those essential properties of its nature which always exist in proportion to the specific quantity of matter so modified. If the immaterial spirits of heat and electricity, as supposed to exist by Dr. Burnett, result from any modification of matter, it is certain that these spirits could not have existed previously to the existence of that modification from which they result; and, if so, these immaterial spirits could not have existed prior to the existence of matter.

The arrangement of materials must necessarily be posterior, in point of time, to the existence of those materials which are thus arranged; and if we admit the pre-existence of those parts which are thus modified, and admit the *immaterial entities* themselves to be the result of a modification which depends upon those parts for its own existence, we behold not only the pre-existence of matter, but the pre-existence even of that *modification* from which these immaterial entities must be supposed to result.

If the "spirits of heat and electricity" result from any given modification of matter, the permanency of that modification is necessary to the existence of these "spirits," which can only result therefrom. To suppose the contrary, destroys the supposition; and to admit the supposition is as repugnant to every principle of philosophy as it is false in fact.

That modification is only an *arrangement of parts* is too evident to admit of contradiction. And to suppose *immaterial entities* to result from a *mere arrangement,* is to suppose that those parts which are thus arranged have communicated to the arrangement of themselves a *potential* quality which they did not possess, and that they have communicated what they could not communicate.

As the *modification* of all material substances can have no positive, but only a relative existence, and can exist no further than as it depends upon matter, so it can, of itself, have no effects. Nothing can result from a *mere relation.* For if a mere relation can produce the *immaterial* entities, heat and electricity, which Dr. Burnett affirms does exist, this relation must be their cause; and, to suppose anything to be a cause, which, of itself, has no positive existence, is to suppose it to act without a being, and that it produces what it has no power of producing.

The *immaterial entities* resulting from matter must still look up to matter as their remote cause; and whether we suppose *immateriality* to be the remote, or the immediate result of matter, it must either be a necessary effect, or an accident of it. To suppose it to be a necessary effect, is to make a quality to result from matter with which it (matter) can have no relation; and, to suppose immateriality to be an accident of it, is to destroy the *necessity* of any peculiar modification of matter in order to its existence.

Thus, then, consider these immaterial entities—heat, electricity, magnetism, &c.—in what relation soever we may to matter, it ends either in an absurdity or a contradiction; and in no case to which Dr.

Burnett has alluded, can any such relation be made out as is necessary to establish that connexion between these *immaterial entities* and matter which must ever subsist between an effect and its cause.

To illustrate, however, more clearly, the apparent probability of the positive existence of these immaterial substances, the author brings in review before us the creation; and claims the privilege of Ralpho, who—

> "Profest
> He had First Matter seen undrest:
> He took her naked, all alone,
> Before one rag of form was on:"

while he describes the *modus operandi* of those immaterial agencies which, he affirms, have contributed to the present shape, order, and beauty that we find impressed upon the world around us.

This account of the world's creation implies, 1st, the previous existence of the immaterial substances in question; and 2ndly, the fact, that the properties which we see associated with matter, depend solely on the superaddition of these immaterial substances.

"It is most strikingly remarkable," observes Dr. Burnett, "that, at the very opening of revelation it should be stated that, in the beginning, when God created the earth, it was 'without form and void, and darkness was upon the face of the deep,' *till* the Spirit of God moved upon the waters. *After* this event, God said 'Let there be light!' He divided the waters from above and from under the firmament, gathering the latter together to form the solid ground. All this he did by his Spirit causing the great immaterial causes of heat and light, electricity and magnetism, to act upon material substance which previously was void and shapeless, uncombined, and probably in a gaseous state, but which he had created distinct from the immaterial substances. We must not lose sight of the fact I have already stated, of these *great immaterial substances* having been in the first instance like the material elements, brought into existence at some previous time before they were employed by the Creator in the original formation of the earth, when it received its first shape, order, and beauty at his hands. And, I would add, is it possible that mere bodies, alike in nature to the chaos that was acted upon, were the only instruments of this wonderful creation."—pp. 55, 56.

It is beyond doubt, that our author regards the existence of materiality as altogether dependent on the superaddition of what he terms the great immaterial causes of heat and light, electricity and magnetism.

It has been already shown that these supposed immaterial causes do not possess an abstract positive existence, that matter does not owe its primary constitution to their influence or co-existence, that modifications of matter cannot originate qualities opposite to its nature,—and we shall now further demonstrate, that neither can the existence of matter, nor any of its essential properties, be in any way dependent on their superaddition.

Heat, light, electricity, and magnetism, are but qualities — the qualities of matter, regarded as *secondary* only; admitting that they had a being anterior to matter, the same distinction will apply. But we have no hesitation in asserting that, *mere qualities considered*, as such, cannot possibly have an abstract existence. Whatever is a quality must be a quality of some substance; the mind is necessarily obliged to associate together the two ideas. To suppose anything to be a quality, without admitting the existence of some substance of which it is the quality, is a contradiction; it supposes it to be a quality and not a quality at the same time.

If heat, light, electricity, magnetism, &c., be qualities superadded to matter, as Dr. Burnett affirms, we would ask,—Of what are these the qualities? They must be of matter, or they must not. If they be qualities of matter, matter must be their cause, and if so, they cease to be *superadded;* if not, their existence is thus ascertained distinct from matter; and the mind, in order to find their substances, is led to explore another source.

If heat, electricity, &c., be qualities *superadded* to matter, these, as well as matter, must have existed antecedent to their union with each other. So far we agree with Dr. Burnett. Matter must have existed previous to the accession of these qualities, for qualities could not be added to that which did not exist. Heat, electricity, magnetism and light, must have existed also, or they could not have been *communicated* to matter. Existence must always be previous to any modification of it. And if matter, heat, electricity, &c., exist prior to their union with each other, it then follows, that this new accession of qualities in matter—the previous existence of which has been admitted—does not depend for its existence upon their union with matter. And if this dependence be taken away, it must also follow, that heat, electricity, light, and magnetism, &c., may as well exist after their separation from matter, as they did before their union with it. Either these qualities must have *existed* prior to their union with matter—which Dr. Burnett maintains—or they must not. If they did, they cannot be qualities of matter; if they did not, they cannot be superadded.

The author's views on life, instinct, and mind, in their normal and abnormal relations, next claim our notice.

"It will be seen," he says, "that the spirit of life in its simplest manifestation in the structures and functions of plants, is a spirit that has the power of PUTTING TOGETHER the primary elements, and particularly the gaseous elements of materiality, in such a manner and in such unions as are nowhere to be traced in bodies that are without this spirit."—p. 100.

That which possesses the power of *putting together* the primary elements of materiality must necessarily be something distinct, and independent of the materials on which it operates—this *something* Dr. Burnett denominates the immaterial spirit of life!

Finding himself, however, unable to reconcile the phenomena of life on the hypothesis of an abstract self-subsistent immaterial entity—he observes:—

"That what is termed life and mind are modes of action resulting from the application of immaterial substances of a higher order to inorganic matter, by which means new combinations are formed, which constitute the material basis of living bodies.

"It is, therefore, incorrect to speak of life as exclusively of an immaterial nature or even character, because that term is made use of to express phenomena, the result of the mixed application of spiritual to material substances.

"Life, therefore, is not a material nor an immaterial entity, but like light and heat, it is only a mode of action produced in the manner I have stated, and mind is a similar mode of action. Life and mind, then, like light and heat, are modes of action resulting from the concurrence of the two grand classes of entity we have been considering." —p. 102.

The author ascribes to *life*, first, an abstract self-subsistency—next, that it is neither material nor immaterial—and, lastly, that it is a mode of action. How this can be, we are at a loss to divine? If existence can be predicated of life—that existence must be either *real* and *absolute*, or *relative* and *dependent*—material or immaterial. If life be a spirit, it must have a positive existence; and not being matter, is necessarily immaterial. There are but two primary substances in the vast empire of created nature, which have in themselves a positive existence; and these are, *matter* and *spirit*. To assert, then, that life is neither a material nor an immaterial entity, after having assigned to it the properties and powers of an agency absolutely immaterial—"a spirit that has the power of *putting together* the primary elements, and particularly the gaseous elements of materiality"—is contradictory and absurd. The author further observes:—

"It is, then, very palpable that the spirit of life *has a power of controlling* those spirits we have shown to possess so wide a power over inorganic matter."—p. 103.

"The effect of the spirit of life upon material substances is again too remarkable to be readily confounded with any other efficient spiritual cause; and in comparing its phenomena with those that are associated with it in the complex machinery of life, we may trace the offices and powers of the spirits it regulates, as of those that control it."—p. 131.

"There is another circumstance in connexion with the spirit of life which is very remarkable, as showing that this spirit is one *sui generis*. I would allude now to the apparently latent state in which the spirit of life is retained in the seeds of vegetables, whose delicate structures rapidly perish when this spirit is removed."—p. 136.

These passages clearly show that Dr. Burnett regards life as a distinct spiritual entity. An opinion which throws no additional light on the mysterious subject to which it refers, while it differs in the words only, from the "Archæus" of Paracelsus and Van Helmont—the "Anima" of Stahl—the "Vis Conservatrix" and "Vis Medicatrix Naturæ" of Hoffman and Cullen—and the "Vital Principle" of some modern physiologists—notions more fanciful than real, and long since exploded.

Life is the *property* of organized structures; and we can no more explain this property, apart from a consideration of those structures which manifest it, than we can any property of inorganic matter, apart from the matter in which it inheres.

There can be no manifestation of animal life apart from respiration, circulation, digestion, assimilation, and excretion—the *dynamics* of matter peculiarly combined.

These processes, so wonderful, and seemingly so complex, are nothing more than refined illustrations of combustion, mechanical force, chemical solution, and filtration.

The attempt which Dr. Burnett has made to explain the various organic functions, on the supposition that a distinct and peculiar spiritual entity reigns with sovereign power in the economy, is altogether fallacious and absurd.

We would ask Dr. Burnett to explain how this unknown something, this separate single entity, is able to govern so many and diverse operations? One secretion differs from another in composition and property—and every part of the organism varies in composition, appearance, and size—while these dissimilar fluids and structures are obtained directly from the blood. If the "spirit of life," according to the explanation which the author gives of it, be the agent, by what show of reasoning can it be made to appear, that the *same cause* can produce such a *variety of effect?*

According to this doctrine, it either must be admitted that there is a different "spirit of life" for every organ—one for the lachrymal glands, another for the liver, a third for the pancreas, &c.—and, indeed, for every form of combination; or, if we grant a power so *varied* and *selective* to a single entity, we distinctly give to it the character of the soul, and endow it with volition and consciousness. But we cannot *will* the circulation of the blood, nor are we *conscious* of the secretion of bile.

Further, the author has not omitted to explain how the "spirit of life" maintains the *integrity of the organized structures*, or, in other words, the phenomena of *healthy function.*

"The office of this spirit," he observes, "is to charge the materials brought together, assimilated and united as they are in the operations of the chylopoietic viscera, with such power as that they may continually repair and build up those parts which otherwise would be destroyed by the different processes going on of chemical change and decomposition. It thus furnishes and controls, in every organ of the body, the several powers of secretion, formation, and growth, in the accomplishment of which it engages the spirits of heat and electricity." —p. 152.

The "*spirit* of life" being thus characterized, it cannot therefore be made available to the elucidation of *disordered function.* If so, then this immaterial entity is itself diseased, and also its coadjutors, the spirits of heat and electricity. But if this will not be conceded, it must be acknowledged that a deterioration of matter, which Dr. Burnett pronounces to be incapable of existence apart from heat and electricity, has been the cause of *the derangement of its only source of activity.*

To diseased structure, then, must we refer the disordered function which accompanies it, just in the same manner that we refer to the integrity of parts the healthy properties which they manifest. So peculiar, indeed, are the abnormal functions of the body, that if we concede a "spirit of life," or, in other words, a *spirit of health,* having the powers which have been ascribed to it, we must necessarily acknowledge a *separate spirit of disease.* The action which at one time generates sound tissue, at another time, by its excess simply, generates morbid tissue. Thus, the "spirit of life"—admitting it to exist—which "controls in every organ of the body the several powers of secretion, formation, and growth," and therefore *a cause of health* plainly becomes a source of disease. If this cause be a "spirit of life," in the sense intended by Dr. Burnett, wherefore is it that this spirit destroys the uses of the parts it is acknowledged to be only concerned in creating, preserving, and defending?

Again, the theory of Dr. Burnett not only fails to explain the phenomena of life and health, but leaves us quite at a loss to understand the phenomena of disease and death.

We are accustomed to regard *dissolution* as dependent upon certain organic changes resulting from the altered arrangements and dispositions of matter, for which we believe the common tendencies and affinities of material particles, *under peculiar circumstances,* to account sufficiently. But if the matter which composes our organism be entirely under the dominion of the "spirit of life," co-operating with the spirits of heat and electricity,—be subject to its exclusive authority and influence, or, in the words of Dr. Burnett, "furnishes and controls in every organ of the body the several powers of secretion, formation, and growth," and this agent be itself immaterial, intangible, and, therefore, by any natural body invulnerable,—there should be no such thing as corporeal decay, or loss of life by any other means apart from external violence! If a "*spirit of life*" have the exclusive government of the organic fabric, *with creative, conservative, and reparative powers and tendency,* death ought never to occur except on the complete disintegration of the material fabric, by chemical or mechanical force.

Life, then, is the *manifested property* of living tissues,—it is that *something* that belongs to matter only *in certain states,* of which we are, for the past, ignorant; and we believe that *every form of matter capable of organization may exhibit the most elaborate function when placed in the circumstances appropriate to its development.*

And the reason that organic actions are not imitable by us to the same extent as are inorganic, depends upon the fact that *vital actions* can only be exercised under conditions which a living being supplies, and of which we cannot avail ourselves.

The organized structures do not change the properties of the elements of which they consist, but simply combine them in modes beyond our capability to imitate.

The action by which vitality is manifested, is a property of matter in a state of organization—a *consequence* of organization, and not a *cause* of it—and no matter can be brought into an organized condition without displaying the phenomena of life: vitality is not the cause of vital action, but the character of the being which exhibits such action.

If the organism of animals be dependent on the "spirit of life" for the manifestation of vital phenomena, every process performed by living materiality depends upon this spirit; in fact, the necessity of *structure* is altogether destroyed. Many of the processes peculiar to plants and animals, that were once believed to be regulated by a separate vital force, are now known to be entirely dependent upon structure, and to be obedient to physical laws, which act *under conditions supplied by the living system.*

Thus the functions of absorption and transudation, both in the animal and the vegetable kingdom, are chiefly due to capillary attraction, and to the phenomena of *endosmose* and *exosmose.*

The arrangement and disposition of ultimate atoms, from which vitality springs, can only be communicated by a living being; that is, by parent to offspring.

Hence, in our inquiry into the efficient cause of vital endowment and property, we are necessarily carried back to the period when the Creator thought fit to collect the "dust of the earth," and to give to it a function not possessed by its fellow dust, that He might be honoured and glorified, not less in the variety than in the unity of His works. The property of life having been imparted to matter, it was decreed that it should continue its action from generation, just as the earth, and the heavens at this moment, revolve in obedience to the forces of attraction and repulsion, which in the beginning guided and governed their movements.

It may be urged, that if the "spirit of life" does not exist nor vitality depend upon it, what induces the peculiar arrangement of material particles from which vitality results? This question is totally unanswerable. And so are hundreds of others, in connexion with physical phenomena. Who can explain how four elements, combined in one proportion, form *bread*, in another *meat*, in a third *opium*, &c.? or to what cause is to be referred the fact, that the *tasteless sap*, which rises in the peach-tree, should produce in the kernel of the fruit a poison—in that of the palm-tree a nutritive food? These *facts* are before us. They do not admit explanation. They constitute proofs that there is a Being all-*powerful* and good, "*in whom we live, move, and have our being.*" We must, however, reserve the conclusion of our analysis for another number.

